# Novel Multi-AP Coordinated Transmission Scheme for 7th Generation WLAN 802.11be

**DOI:** 10.3390/e22121426

**Published:** 2020-12-17

**Authors:** Woojin Ahn

**Affiliations:** Train Control & Communication Research Team, Korea Railroad Research Institute (KRRI), Uiwang-si 16105, Korea; woojin.ahn@krri.re.kr; Tel.: +82-31-460-5784

**Keywords:** WLAN, channel access, triggered uplink access, multi-AP coordination

## Abstract

The demand for high-data-rate and time-sensitive applications, such as 4k/8k video streaming and real-time augmented reality (AR), virtual reality (VR), and gaming, has increased significantly. Addressing the inefficiency of distributed channel access and the fairness problem between uplink and downlink flows is crucial for the development of wireless local area network (WLAN) technologies. In this study, we propose a novel transmission scheme for IEEE 802.11be networks that addresses the fairness problem and improves the system throughput. Utilizing the concept of multi-AP coordinated OFDMA introduced in the 7th-generation WLAN IEEE 802.11be, the proposed transmission scheme allows an AP to share a granted transmission opportunity (TXOP) with nearby APs. A mathematically analysis of the throughput performance of the proposed schemes was performed using a Markov chain model. The simulation results verify that the scheme effectively improves the downlink fairness and the system throughput. Combined with the advanced multiuser (MU) features of IEEE 802.11ax, such as TUA, MU cascading sequence, and MU EDCA, the proposed scheme not only enhances downlink AP transmission, but also guarantees improved control over the medium. The scheme is carefully designed to be fully compatible with conventional IEEE 802.11 protocols, and is thus potentially universal.

## 1. Introduction

IEEE 802.11 wireless local area network (WLAN) technology is the standard protocol used to implement high-speed wireless connectivity, especially for indoor devices, owing to its ease of deployment and cost efficiency. As reported in [1], more than 9 billion Wi-Fi devices are currently in use worldwide, and Wi-Fi-based technologies were valued at approximately USD 2 trillion as of 2018. The market value of the Wi-Fi industry is estimated to reach USD 3.5 trillion by 2023. Moreover, the number of connected devices is growing at an astounding rate of 10% compound annual growth rate (CAGR), compared with the global population and number of Internet users, which are growing at 1.0% and 6% CAGR, respectively. This has resulted in a significant increase in the average number of stations and connections per network [2].

Overlapping WLAN zones with greater station density degrade the overall network performance significantly, owing to the inefficiency in channel usage inherent to WLAN protocols. Previously employed standard protocols witnessed a significant increase in nominal data rates, but such a trend has not been observed for WLAN medium access control protocols. As the channel contention overhead increases exponentially with the network density [3], inefficiency in dense network environments is a major drawback of the technology.

Furthermore, the distributed coordination function applied in most WLAN devices as the primary channel access function allows all devices to have the same channel access probability. It has been demonstrated that that uplink (UL) flows dominate the downlink (DL) flows in access points (APs) [4,5,6]. To address the medium access efficiency problem in high-data-rate and time-sensitive applications, such as 4k/8k video streaming, real-time AR, VR, and gaming, the latest WLAN standards IEEE 802.11ax (high efficiency WLAN), and the potential successor, IEEE 802.11be (extremely high throughput WLAN) focus on improving the fairness of APs.

The IEEE 802.11ax standard, also known as Wi-Fi 6, has been employed since 2014. The main objective of IEEE 802.11ax is to achieve high efficiency in dense network environments [7]. By introducing a revolutionary feature called the orthogonal frequency division multiple access (OFDMA), IEEE 802.11ax enables multiuser channel access and concurrent transmissions with high flexibility, by grouping the OFDMA subcarriers into non-overlapping resource units (RU). In addition, the IEEE 802.11ax standard incorporates schemes such as triggered UL access (TUA) and multiuser enhanced distributed channel access (MU EDCA) to address the CSMA congestion problem. This leads to APs having prioritized control on the medium. Using TUA, APs can trigger multiplexed UL transmission from multiple stations simultaneously, using various resource domains (e.g., the frequency or spatial domain). Moreover, MU EDCA prioritizes the channel access intensity of APs over stations by differentiating the channel access parameters, enabling APs to trigger more multiplexed transmissions.

The IEEE 802.11be [8,9] task group was established in May 2019. The protocols include a new concept of collaboration among neighboring 802.11be APs and multi-AP coordination, to realize efficient utilization of resources among different basic service sets (BSSs). Among several conceptual implementations of multi-AP coordination, coordinated OFDMA is one that mitigates the collision probability more effectively than in the case when APs perform independent channel access. This is because OFDMA allows APs to synchronize data transmissions using orthogonal time and frequency resources.

Several approaches based on conventional IEEE 802.11 standards have been proposed to address the medium access efficiency problem. The approach adopted in [4] prioritized APs by assigning smaller contention windows (CW). Although this approach yields a higher AP throughput, a higher number of collisions degrades the system reliability. The authors of [5,10] proposed assigning higher TXOP limit values to APs to aggregate more DL data. This approach is beneficial in reducing the contention overhead, but it may result in relatively starved UL flows as the number of stations increases. In [6], a bidirectional transmission scheme that increases DL transmissions significantly was proposed, using the reverse direction protocol (RDP) defined in [11]. However, it has the following drawbacks: DL transmissions may not occur in the reverse direction when APs have no queued data from the RDP initiating station, and the throughput variance among stations can increase.

To address the aforementioned problems, in this study, we propose a novel transmission scheme for IEEE 802.11be networks that improves the fairness between UL and DL, along with the system throughput. Utilizing the concept of the multi-AP coordinated OFDMA, the proposed transmission scheme follows the conventional IEEE 802.11 channel bonding rule to allow APs to share granted transmission opportunities (TXOPs) with other APs in coordinated AP sets, whenever a wide-band transmission is possible. Without the need for a complicated coordination or reservation process, APs may participate in coordinated transmissions opportunistically in response to spontaneous triggering frames from other APs in the set.

The remainder of this paper is organized as follows. In Section 2, the proposed scheme is described in detail, including the background technologies. In Section 3, a mathematical analysis using Markov chain analysis is presented. The simulation results of the proposed scheme are presented in Section 4, and the concluding remarks are provided in Section 5.

## 2. Proposed Scheme

In this study, a novel transmission scheme utilizing the concept of multi-AP coordinated OFDMA of IEEE 802.11be. The proposed scheme is designed to take full advantage of advanced multiuser (MU) features of IEEE 802.11ax.

**Figure 1:**
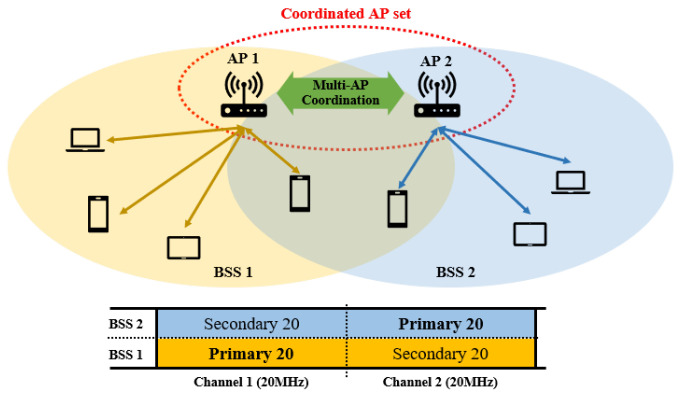
An example of multi-AP coordinated transmission scenario.

As illustrated in Figure 1, we consider a scenario where two APs of adjacent WLAN BSSs are within each other’s range of transmission, thereby belonging to a potential coordinated AP set. We assume that one of the secondary channels of a BSS is overlapped with the primary channel of the other. The two APs can exchange frames for coordinated transmission by extending the transmission bandwidth, following the conventional channel bonding policy of the IEEE 802.11 standards.

### 2.1. MU Features in IEEE 802.11ax

In addition to conventional EDCA, IEEE 802.11ax station can access the medium whenever a TUA procedure is triggered. A TUA procedure is initiated when an AP transmits a trigger frame (TF), which ensures that multiple STAs are allowed to transmit data in response to the TF. The main purpose of the TF is to share time synchronization information among scheduled stations, in addition to scheduling information for each station. It is designed to have multiple variants for various purposes.

IEEE 802.11ax also introduces two novel features based on TUA.

An MU cascading sequence is a frame exchange sequence between an AP and one or more associated stations, and is characterized by the exchange of frames in both directions in a single TXOP. In an MU cascading sequence, the AP initiates its TXOP by transmitting DL MU data in the form of an MU physical-layer protocol data unit (PPDU). In general DL MU transmission, the AP triggers a subsequent UL MU transmission using TF, to obtain acknowledgments from multiple recipients. Since a DL MU recipient can only decode data located in the scheduled resource unit (RU), the AP piggybacks a unicast TF with the DL data. In order to trigger an MU cascading sequence, the AP can indicate extended UL MU transmission duration through the piggybacked TF, to ensure that UL data can also be aggregated together with the acknowledgment. In this case, following the TUA behavior of the IEEE 802.11ax standard, multiple stations perform UL MU transmission SIFS after the received (DL) MU PPDU, using the trigger-based (TB) PPDU format. At the end of the sequence, the AP transmits a multi-STA BlockAck frame defined in the IEEE 802.11ax, in response to the UL data from multiple stations.

The main benefit of the MU cascading sequence is that the channel utilization of TXOPs can be improved by soliciting multiplexed transmission bidirectionally without incurring additional channel access. The improved multiplexing gain of AP, however, may not necessarily lead to a remarkable throughput enhancement. This is because the channel access intensity, which is mainly affected by the EDCA parameters (e.g., contention window parameters) of the AP is the same as that of an individual station. The chance for the AP to obtain a successful TXOP decreases severely as the number of associated stations increases. Considering this aspect, the second additional feature, MU EDCA, can play an important role in combination with the MU cascading sequence, especially in dense network environments.

**Figure 2:**
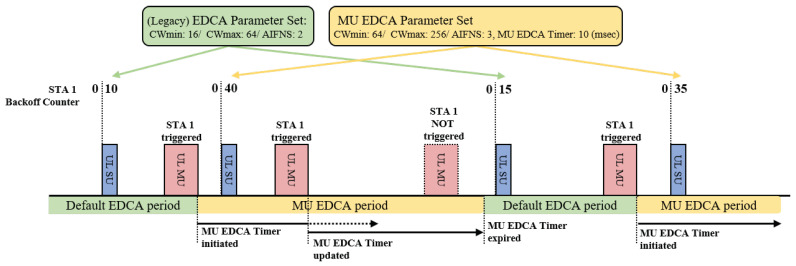
An example of MU EDCA protocol.

MU EDCA is a novel protocol according to which APs lead associated stations to use an alternate set of EDCA parameters, called the MU EDCA parameter set. This set includes contention window parameters and a countdown timer (MU EDCA timer) for each access category, with the objective of lowering the channel access probability of non-AP stations. When a station successfully transmits UL data for an access category in response to a TF, the station sets its EDCA parameters to the values of the MU EDCA parameter set for the corresponding access category, and begins the countdown from the MU EDCA timer value to zero. If the station succeeds in sending another UL MU transmission before the timer expires, it resets the timer to the MU EDCA timer value. If the timer expires, it sets its EDCA parameters to the values of the (legacy) EDCA parameter set, as illustrated in Figure 2. By setting the MU parameter set values to be greater than the EDCA parameter set values, APs can achieve more prioritized channel access probabilities. To retain stations in the MU EDCA mode, the AP needs to trigger UL MU transmission periodically to ensure that the countdown timers of the stations extend consistently.

### 2.2. Proposed Multi-AP Coordinated Transmission Scheme

**Figure 3:**
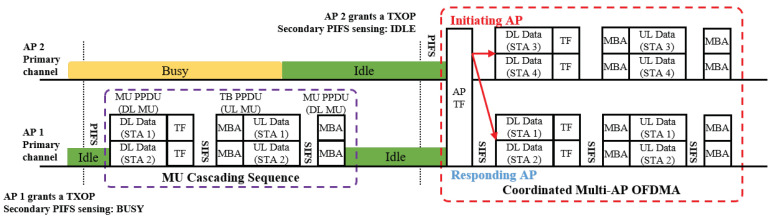
An example of the proposed multi-AP coordinated transmission sequence.

**Figure 4:**
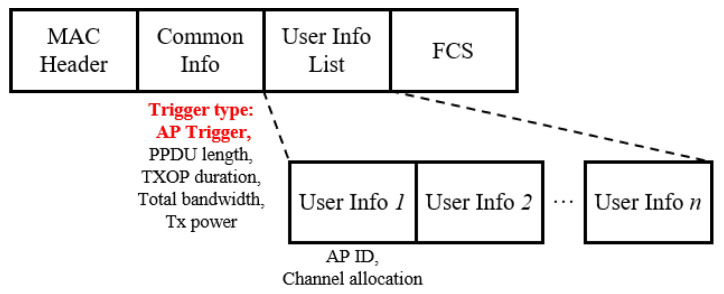
The general format of ATF.

In the proposed scheme, TF is used to solicit a nearby AP to initiate a coordinated transmission sequence. The general transmission sequence of the proposed scheme is illustrated in Figure 3. Whenever an AP acquires a TXOP after completing its backoff procedure, it may perform a wide-band transmission if the secondary channel is idle during the PCF interframe spacing (PIFS) preceding the transmission. If wide-band transmission is allowed, the AP may choose to transmit an AP trigger frame (ATF), a variant of TF where the general format is depicted as shown in Figure 4, to other APs (responding APs) in the same coordinated AP set. This is considered the initiating AP, with the primary channel being occupied by the wide-band transmission. In the proposed coordinated transmission sequence, the initiating AP assigns one or more of the secondary channels to one of the responding APs. In this case, the assigned channel should include the primary channel of the responding AP. The ATF contains information related to the coordinated transmission that immediately follows the ATF, such as the duration of the TXOP, the bandwidth of the TXOP, and the scheduled channel information. Since all participating APs use mutually exclusive channels, the proposed scheme does not require any scheduling information on the responding APs. Moreover, each AP utilizes the scheduled channel exclusively, thus acting as the TXOP holder for the corresponding channel.

The channel access behavior of the proposed coordinated transmission scheme is the same as the general TUA behavior, as defined in [12]. Upon reception of the ATF, the initiating and responding APs start simultaneous DL transmission short interframe spacing (SIFS) after the ATF. In this case, all transmitting APs should perform energy detection (ED) clear channel assessment during the SIFS period. As the backoff procedure for granting the wide-band TXOP is performed solely by the initiating AP, all responding APs should terminate the entire transmission sequence before the TXOP duration, which is indicated by the received ATF. Furthermore, all responding APs transmit data as a TXOP responder, and the backoff procedure of the responding APs is not affected by the transmission. The responding APs resume their backoff procedure with the remaining backoff counter. Therefore, the greater the number of coordinated transmissions, the greater the channel access intensity of the APs.

In addition, two novel features adopted from IEEE 802.11ax, MU cascading and MU EDCA, maximize the efficiency of the proposed coordinated transmission.

Combined with MU cascading, AP can greatly increase its per TXOP transmission efficiency of the proposed scheme. In response to an ATF, as shown in Figure 3, both the initiating and the responding AP can piggyback a TF in their DL MU PPDU to solicit a subsequent UL MU transmission. In this case, each piggybacked TF contains scheduling information for the stations associated to the transmitting AP. The scheduled RUs of both APs do not overlap and includes their primary channel so that a complicated resource coordination can be avoided.

MU EDCA helps the proposed scheme to greatly increase chances to trigger the AP coordination sequence. Since MU EDCA can be applied to the TF piggybacked in the DL MU transmission of the proposed MU cascading sequence, stations can stay in the MU EDCA mode without APs acquiring additional TXOPs for TF transmission.

In the next section, a mathematical model for the analysis of the proposed scheme is provided.

## 3. Numerical Analysis

In this section, we present a numerical analysis of the proposed scheme. The purpose of this analysis is to obtain the system throughput of the proposed multi-AP coordinated transmission scheme. We assume two adjacent BSSs belonging to the same coordinated AP set, each with operation bandwidths of 40 MHz, and the 20 MHz primary channel of one of the BSSs overlaps with the 20 MHz secondary channel of each other, as illustrated in Figure 1. In each BSS, a fixed number of stations are associated with the AP, and both the AP and the stations have saturated AC_VO traffics with the same payload sizes, *l*. We assume ideal channel conditions for the analysis. Thus, only the effect of packet collision is considered, and the capture effect [13] is neglected. Given these assumptions, the AP and the stations in each BSS perform EDCA on their primary channels and transmit a wide-band PPDU whenever a wide band transmission is allowed. The APs are configured to initiate the proposed coordinated multi-AP transmission sequence for wide-band transmission. Otherwise, a narrow-band MU cascading sequence is initiated without multi-AP coordination.

Markov chain analysis [3] is one of the most popular methods for obtaining the system throughput of WLAN networks. A virtual slot is the time interval between two consecutive backoff counter decrements of nontransmitting stations. Since the capture event is not considered, all stations in the same BSS are synchronized by virtual slots occupying the primary channel. In addition, a wide-band virtual slot temporarily synchronizes BSSs belonging to the same coordinated AP set. Therefore, it is necessary to understand the stochastic properties of wide-band transmission events.

**Figure 5:**
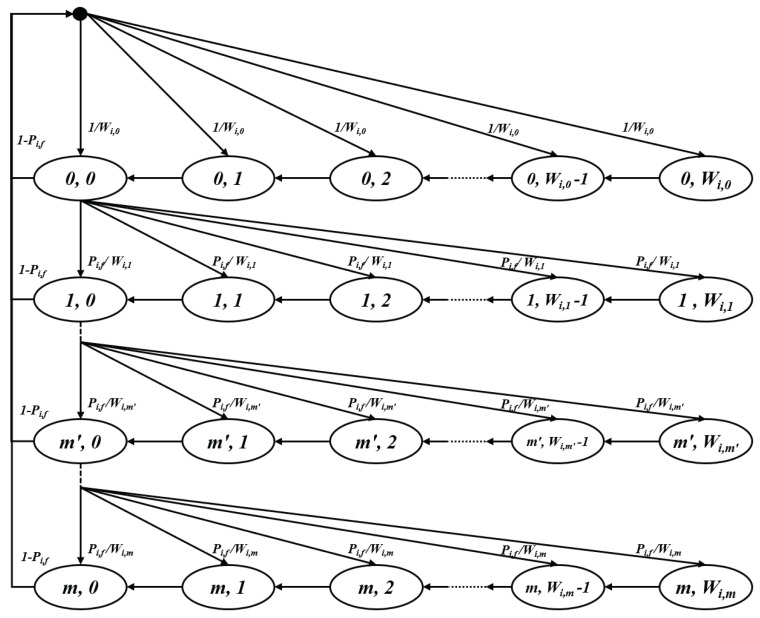
Markov chain backoff procedure.

Since APs in target BSSs use single EDCA parameter sets, the Markov chain model of APs with saturated traffic conditions is based on the two-dimensional Markov chain model described in [3]. For non-AP stations, if we assume that the MU EDCA timer value is large enough such that APs can trigger the same stations before the timers expire, the steady-state Markov chain model of stations also follows the same model with different EDCA parameters. Figure 5 depicts the Markov chain model of a device, where *i* is the target BSS following the proposed scheme, and $W_{i,0}$, $W_{i,m^{\prime }}$, *m*, and $P_{i,f}$ denote the minimum contention window size, maximum contention window size, retry limit, and probability of transmission failure, respectively. Since a lossless channel is assumed, a transmission failure is caused only by the collision in the corresponding slot.

(1) $$W_{i,n}=\{\begin{array}{cc} 2^{n}W_{i,0} & n≦m^{\prime }\\ 2^{m^{\prime }}W_{i,0} & m^{\prime }<n≦m \end{array}$$

After solving the balance equations of the Markov chain model, the stationary probability distribution can be obtained according to (2) and (3).

(2) $$\left\{ \begin{array}{cc} \begin{array}{c} b_{i,n,k} \end{array} & =\frac{W_{i,n}-k}{W_{i,n}}\\ \; & =\{\begin{array}{cc} \frac{2^{n}W_{i,0}-k}{2^{n}W_{i,0}}b_{i,n,0}, & 0≦n≦m^{\prime },1<k≦W_{i,n}-1\\ \frac{2^{m^{\prime }}W_{i,0}-k}{2^{m^{\prime }}W_{i,0}}b_{i,n,0}, & m^{\prime }≦n≦m,1<k≦W_{i,n}-1 \end{array}\\ b_{i,n,0} & =P_{i,f}^{n}b_{i,0,0},0<n≦m \end{array}\right.$$

(3) $$b_{i,0,0}=\{\begin{array}{cc} {\left[\frac{W_{i,0}\left[1-{\left(2P_{i,f}\right)}^{m+1}\right]}{2(1-2P_{i,f})}-\frac{1-P_{i,f}^{m+1}}{2(1-P_{i,f})}+\frac{1-P_{i,f}^{m+1}}{1-P_{i,f}}\right]}^{-1} & ,m≦m^{\prime }\\ {\left[\frac{W_{i,0}\left[1-{\left(2P_{i,f}\right)}^{m^{\prime }+1}\right]}{2(1-2P_{i,f})}-\frac{2^{m^{\prime }}W_{i,0}\left(P_{i,f}^{m^{\prime }+1}-P_{i,f}^{m+1}\right)+P_{i,f}^{m+1}-1}{2(1-P_{i,f})}+\frac{1-P_{i,f}^{m+1}}{1-P_{i,f}}\right]}^{-1} & ,m>m^{\prime } \end{array}$$

Let $\tau_{i}$ be the probability that a device, *i*, transmits in a randomly chosen virtual slot by an EDCA contention. Since an EDCA transmission occurs when the backoff counter reaches zero, regardless of the backoff stage, $\tau_{i}$ is expressed as a function of $b_{i,0,0}$ and $P_{i,f}$ as follows:

(4) $$\tau_{i}=\sum_{n=0}^{m}b_{i,n,0}=\sum_{n=0}^{m}P_{i,f}^{n}b_{i,0,0}=\frac{1-P_{i,f}^{m+1}}{1-P_{i,f}}b_{i,0,0}$$

As the system throughput, *S*, can be defined as

(5) $$S=\frac{E[\mathrm{payload}\mathrm{bits}\mathrm{transmitted}\mathrm{in}a\mathrm{virtual}\mathrm{slot}]}{E[\mathrm{duration}\mathrm{of}a\mathrm{virtual}\mathrm{slot}]}=\frac{E[L]}{E[T]},$$

All possible virtual slots need to be specified, along with the corresponding event probabilities. These can be derived using the EDCA transmission probability of the AP and the stations in the target BSS ($\tau_{a}$ and $\tau_{s}$), as well as the EDCA transmission probability of the AP and the stations in the BSS pair ($\tau_{a^{\prime }}$ and $\tau_{s^{\prime }}$).

Before deriving all virtual slots of the proposed scheme in detail, we define several mathematical expressions that are frequently used in the latter part of the analysis. The following expressions correspond to the probability of virtual slots of an isolated BSS with a single operating channel. $P_{idle}$ denotes the probability that a given slot is an idle slot where no device attempts to transmit. $P_{a,suc}$, and $P_{s,suc}$ are the probabilities of EDCA transmission success of the AP and the station, respectively. $P_{a,col}$ denotes the probability that an EDCA transmission of the AP collides with other EDCA transmissions of stations, and $P_{s,col}$ denotes the probability that EDCA transmission of stations collide without transmission of the AP. Assuming that the number of stations in the target BSS is *N*, we have:

(6) $$\left\{ \begin{array}{c} P_{idle}=\left(1-\tau_{a}\right){\left(1-\tau_{s}\right)}^{N}\\ P_{a,suc}=\tau_{a}{\left(1-\tau_{s}\right)}^{N}\\ P_{s,suc}=N\tau_{s}\left(1-\tau_{a}\right){\left(1-\tau_{s}\right)}^{N-1}\\ P_{a,col}=\tau_{a}\left[1-{\left(1-\tau_{s}\right)}^{N}\right]\\ P_{s,col}=\left(1-\tau_{a}\right)\left[1-{\left(1-\tau_{s}\right)}^{N}-N\tau_{s}{\left(1-\tau_{s}\right)}^{N-1}\right] \end{array}\right.$$

To simplify the analysis, if we assume that the number of stations in the pair BSS, $N^{\prime }$, is equal to *N* and that all EDCA parameters of the BSSs in the same coordinated AP set are equal, equivalent expressions for the pair BSS can be obtained as follows:

(7) $$\left\{ \begin{array}{c} P_{idle^{\prime }}=\left(1-\tau_{a}^{\prime }\right){\left(1-\tau_{s^{\prime }}\right)}^{N^{\prime }}=P_{idle}\\ P_{a^{\prime },suc}=\tau_{a^{\prime }}{\left(1-\tau_{s^{\prime }}\right)}^{N^{\prime }}=P_{a,suc}\\ P_{s^{\prime },suc}=N^{\prime }\tau_{s^{\prime }}\left(1-\tau_{a^{\prime }}\right){\left(1-\tau_{s^{\prime }}\right)}^{N^{\prime }-1}=P_{s,suc}\\ P_{a^{\prime },col}=\tau_{a^{\prime }}\left[1-{\left(1-\tau_{s^{\prime }}\right)}^{N^{\prime }}\right]=P_{a,col}\\ P_{s^{\prime },col}=\left(1-\tau_{a^{\prime }}\right)\left[1-{\left(1-\tau_{s^{\prime }}\right)}^{N^{\prime }}-N^{\prime }\tau_{s^{\prime }}{\left(1-\tau_{s^{\prime }}\right)}^{N^{\prime }-1}\right]=P_{s,col} \end{array}\right.$$

**Figure 6:**
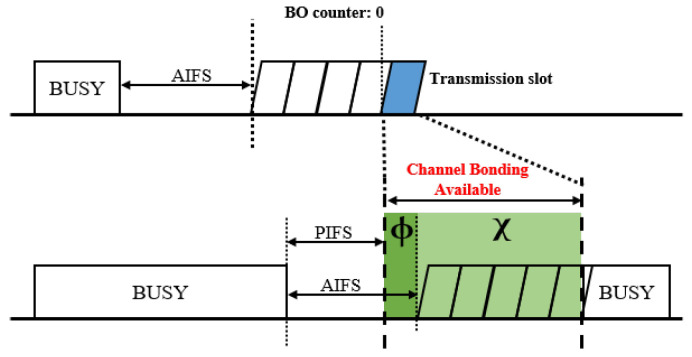
The condition for wide-band transmission.

In the proposed scheme, a coordinated transmission sequence is initiated when the AP attempts an EDCA transmission and the condition for wide-band transmission is satisfied. Therefore, it is necessary to determine the probability of wide-band transmission occurring in each virtual slot. As illustrated in Figure 6, a virtual slot is available for wide-band transmission if the beginning of the virtual slot overlaps with (1) the tail end of the AIFS period following a narrow-band transmission of the other BSS with probability ϕ, or (2) a sensing period with probability χ. Since the duration of AIFS is always longer than that of PIFS, it is possible to interrupt an event slot of the other BSS, where a narrow band transmission occurs. In addition, any idle periods always come after the AIFS deferring a transmission event, and overlapping with an idle period always guarantees that the conditions for wide-band transmission are satisfied. As a result, a virtual slot has a probability of (ϕ+χ) that the condition for wide-band transmission is satisfied.

**Figure 7:**
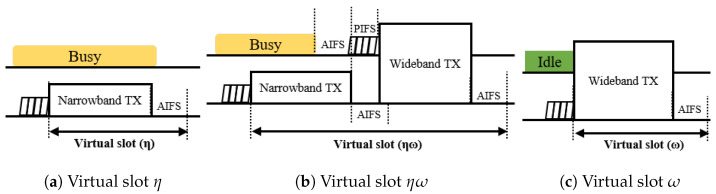
Categories of virtual slots with respect to the event bandwidth.

Considering the wide-band transmission in IEEE 802.11 standards, there are three categories of virtual slots to be defined with respect to the bandwidth of slot events, as depicted in Figure 7: (1) a virtual slot that begins and ends with a narrow band event, denoted by η, (2) a virtual slot that begins with a narrow-band event and ends with a wide-band event, denoted by ηω, and (3) a virtual slot that begins and ends with a wide-band event, denoted by ω.

A virtual slot is denoted by η if the condition for wide-band transmission is not met, with probability (1−χ−ϕ), at the beginning of the slot. As the secondary channel is busy during the preceding PIFS duration, there is no possibility for a device in the pair BSS contending for the medium in wide-band transmission. Taking this into account, there are five possible virtual slots in category η. If there is no transmission among devices in the target BSS, a slot is idle with probability $P_{idle}^{\eta }$. Since the duration of the idle slot is a slot time, δ, which is the smallest time unit of EDCA, the slot ends immediately. The other four types of virtual slots are associated with narrow-band transmission events, and defined as follows: a virtual slot with (1) successful transmission of the AP, (2) a successful transmission of a station, (3) a collision including the AP, and (4) a collision without the AP. Since the AP is configured to start a TXOP with DL MU transmission for a duration longer than the duration of SU transmission for the station, the duration of a collision slot is determined based on whether the AP has transmitted. Regardless of the type of transmission slots, a narrow-band transmission ends with the AIFS deferring. Since the stations of the BSS pair operate with different primary channels, they have different series of virtual slots that are not synchronized to the target BSS. Therefore, if the tail end of the AIFS period of a narrow-band transmission virtual slot overlaps with the channel sensing period of the BSS pair, stations in the BSS pair may interrupt with wide-band transmission. For a narrow-band virtual slot to end without interruption from the BSS pair, the tail end period should not overlap with the sensing period of the pair BSS. Otherwise, none of the devices in the BSS pair attempt to access the channel. According to the default EDCA parameter setting of the IEEE 802.11, the difference between AIFS and AIFS of AC_VO is in the slot time. In this case, there exists only one instance where an interruption from the BSS pair might occur. Summarizing the aforementioned aspects, the probability and duration of the virtual slots in category η can be expressed as

(8) $$\left\{ \begin{array}{cc} \begin{array}{c} P_{idle}^{\eta } \end{array} & =\left(1-\chi -\phi \right)\left(1-\tau_{a}\right){\left(1-\tau_{s}\right)}^{N}\\ \; & =\left(1-\chi -\phi \right)P_{idle}\\ P_{a,suc}^{\eta } & =\left(1-\chi -\phi \right)\tau_{a}{\left(1-\tau_{s}\right)}^{N}\left[\left(1-\chi \right)+\chi \left(1-\tau_{a^{\prime }}\right){\left(1-\tau_{s^{\prime }}\right)}^{N^{\prime }}\right]\\ \; & =\left(1-\chi -\phi \right)P_{a,suc}\left[\left(1-\chi \right)+\chi P_{idle}\right]\\ P_{s,suc}^{\eta } & =\left(1-\chi -\phi \right)N\tau_{s}\left(1-\tau_{a}\right){\left(1-\tau_{s}\right)}^{N-1}\left[\left(1-\chi \right)+\chi \left(1-\tau_{a^{\prime }}\right){\left(1-\tau_{s^{\prime }}\right)}^{N^{\prime }}\right]\\ \; & =\left(1-\chi -\phi \right)P_{s,suc}\left[\left(1-\chi \right)+\chi P_{idle}\right]\\ P_{a,col}^{\eta } & =\left(1-\chi -\phi \right)\tau_{a}\left[1-{\left(1-\tau_{s}\right)}^{N}\right]\left[\left(1-\chi \right)+\chi \left(1-\tau_{a^{\prime }}\right){\left(1-\tau_{s^{\prime }}\right)}^{N^{\prime }}\right]\\ \; & =\left(1-\chi -\phi \right)P_{a,col}\left[\left(1-\chi \right)+\chi P_{idle}\right]\\ P_{s,col}^{\eta } & =\left(1-\chi -\phi \right)\left(1-\tau_{a}\right)\left[1-{\left(1-\tau_{s}\right)}^{N}-N\tau_{s}{\left(1-\tau_{s}\right)}^{N-1}\right]\\ \; & \;\left[\left(1-\chi \right)+\chi \left(1-\tau_{a^{\prime }}\right){\left(1-\tau_{s^{\prime }}\right)}^{N^{\prime }}\right]\\ \; & =\left(1-\chi -\phi \right)P_{s,col}\left[\left(1-\chi \right)+\chi P_{idle}\right] \end{array}\right.$$

(9) $$\left\{ \begin{array}{c} T_{idle}^{\eta }=\delta \\ T_{a,suc}^{\eta }=T_{mu}^{\eta }+T_{tb}^{\eta }+T_{mba}^{\eta }+2T_{sifs}+T_{aifs}\\ T_{s,suc}^{\eta }=T_{su}^{\eta }+T_{ba}^{\eta }+T_{sifs}+T_{aifs}\\ T_{a,col}^{\eta }=\max\left(T_{mu}^{\eta },T_{su}^{\eta }\right)+T_{aifs}=T_{mu}^{\eta }+T_{aifs}\\ T_{s,col}^{\eta }=T_{su}^{\eta }+T_{aifs} \end{array}\right.$$

In (9), $T_{mu}^{\eta }$, $T_{tb}^{\eta }$, $T_{mba}^{\eta }$, $T_{su}^{\eta }$ denote the narrow-band transmission duration of DL MU data, UL MU data, multi-STA BlockAck frame, and UL SU data. $T_{sifs}$ and $T_{aifs}$ denote the durations of SIFS and AIFS, respectively.

If the tail end of a narrow-band transmission is interrupted by a station in the BSS pair, a subsequent wide-band transmission follows. In this case, the devices in the target BSS have not finished AIFS deferring, and cannot decrement their backoff counter. The duration of the virtual slot extends until the end of the subsequent wide-band transmission. In addition, only the devices in the BSS pair can participate in medium contention, and the probability of the subsequent transmission is only affected by the EDCA transmission probability of the devices in the BSS pair. For each type of narrow band transmission event, there are three subtypes of virtual slots: (1) a successful wide-band transmission of the BSS pair AP, (2) a successful wide-band transmission of a BSS pair station, and (3) a collision of wide-band transmissions. For wide-band transmission, the AP is configured to transmit an ATF frame. Therefore, the duration of a collision slot between the AP and a station is determined by the duration of the UL SU transmission under the assumption that the payload size of the UL SU transmission is greater than that of the ATF control information. Since none of the APs or stations in the target BSS can contend the medium during the AIFS period, a collision between the APs does not occur. The probability and duration of the virtual slots in category ηω that follow a successful narrow-band transmission event of the AP in the target BSS can be given by

(10) $$\left\{ \begin{array}{cc} \begin{array}{c} P_{a,suc-a^{\prime },suc}^{\eta \omega } \end{array} & =\left(1-\chi -\phi \right)\tau_{a}{\left(1-\tau_{s}\right)}^{N}\chi \tau_{a^{\prime }}{\left(1-\tau_{s^{\prime }}\right)}^{N^{\prime }}\\ \; & =\chi \left(1-\chi -\phi \right)P_{a,suc}P_{a,suc}\\ P_{a,suc-s^{\prime },suc}^{\eta \omega } & =\left(1-\chi -\phi \right)\tau_{a}{\left(1-\tau_{s}\right)}^{N}\chi N^{\prime }\tau_{s^{\prime }}\left(1-\tau_{a^{\prime }}\right){\left(1-\tau_{s^{\prime }}\right)}^{N^{\prime }-1}\\ \; & =\chi \left(1-\chi -\phi \right)P_{a,suc}P_{s,suc}\\ P_{a,suc-s^{\prime },col}^{\eta \omega } & =\left(1-\chi -\phi \right)\tau_{a}{\left(1-\tau_{s}\right)}^{N}\chi \\ \; & \;\cdot \left\{\tau_{a^{\prime }}\left[1-{\left(1-\tau_{s^{\prime }}\right)}^{N}\right]+\left(1-\tau_{a^{\prime }}\right)\left[1-{\left(1-\tau_{s^{\prime }}\right)}^{N^{\prime }}-N^{\prime }\tau_{s^{\prime }}{\left(1-\tau_{s^{\prime }}\right)}^{N^{\prime }-1}\right]\right\}\\ \; & =\chi \left(1-\chi -\phi \right)P_{a,suc}\left[P_{a,col}+P_{s,col}\right] \end{array}\right.$$

(11) $$\left\{ \begin{array}{c} T_{a,suc-a^{\prime },suc}^{\eta \omega }=T_{mu}^{\eta }+T_{tb}^{\eta }+T_{mba}^{\eta }+T_{atf}^{\omega }+T_{mu}^{\eta }+T_{tb}^{\eta }+T_{mba}^{\eta }+5T_{sifs}+T_{pifs}+T_{aifs}\\ T_{a,suc-s^{\prime },suc}^{\eta \omega }=T_{mu}^{\eta }+T_{tb}^{\eta }+T_{mba}^{\eta }+T_{su}^{\omega }+T_{ba}^{\omega }+3T_{sifs}+T_{pifs}+T_{aifs}\\ T_{a,suc-s^{\prime },col}^{\eta \omega }=T_{mu}^{\eta }+T_{tb}^{\eta }+T_{mba}^{\eta }+T_{su}^{\omega }+2T_{sifs}+T_{pifs}+T_{aifs} \end{array}\right.$$

In Equations (10) and (11), the subscripts denote the sequence of the transmission events. For example, $P_{a,suc-a^{\prime },suc}^{\eta \omega }$ denotes the probability of successful transmission of the AP in the target BSS, followed by successful wide-band transmission of the BSS pair AP in a virtual slot.

When a wide-band transmission of the BSS pair AP is interrupted, the two APs in the coordinated AP set initiate a narrow-band cascading sequence based on their primary channel, once the ATF is transmitted successfully. This is reflected in the calculation of the duration given by (11).

For the other three types of narrow-band transmission events, the interruption of a wide-band transmission from the BSS pair might occur in the same manner. Therefore, we have:

(12) $$\left\{ \begin{array}{c} \begin{array}{c} P_{s,suc-a^{\prime },suc}^{\eta \omega }=\chi \left(1-\chi -\phi \right)P_{s,suc}P_{a,suc} \end{array}\\ P_{s,suc-s^{\prime },suc}^{\eta \omega }=\chi \left(1-\chi -\phi \right)P_{s,suc}P_{s,suc}\\ P_{s,suc-s^{\prime },col}^{\eta \omega }=\chi \left(1-\chi -\phi \right)P_{s,suc}\left[P_{a,col}+P_{s,col}\right]\\ P_{a,col-a^{\prime },suc}^{\eta \omega }=\chi \left(1-\chi -\phi \right)P_{a,col}P_{a,suc}\\ P_{a,col-s^{\prime },suc}^{\eta \omega }=\chi \left(1-\chi -\phi \right)P_{a,col}P_{s,suc}\\ P_{a,col-s^{\prime },col}^{\eta \omega }=\chi \left(1-\chi -\phi \right)P_{a,col}\left[P_{a,col}+P_{s,col}\right]\\ P_{s,col-a^{\prime },suc}^{\eta \omega }=\chi \left(1-\chi -\phi \right)P_{s,col}P_{a,suc}\\ P_{s,col-s^{\prime },suc}^{\eta \omega }=\chi \left(1-\chi -\phi \right)P_{s,col}P_{s,suc}\\ P_{s,col-s^{\prime },col}^{\eta \omega }=\chi \left(1-\chi -\phi \right)P_{s,col}\left[P_{a,col}+P_{s,col}\right] \end{array}\right.$$

(13) $$\left\{ \begin{array}{c} T_{s,suc-a^{\prime },suc}^{\eta \omega }=T_{su}^{\eta }+T_{ba}^{\eta }+T_{atf}^{\omega }+T_{mu}^{\eta }+T_{tb}^{\eta }+T_{mba}^{\eta }+4T_{sifs}+T_{pifs}+T_{aifs}\\ T_{s,suc-s^{\prime },suc}^{\eta \omega }=T_{su}^{\eta }+T_{ba}^{\eta }+T_{su}^{\omega }+T_{ba}^{\omega }+2T_{sifs}+T_{pifs}+T_{aifs}\\ T_{s,suc-s^{\prime },col}^{\eta \omega }=T_{su}^{\eta }+T_{ba}^{\eta }+T_{su}^{\omega }+1T_{sifs}+T_{pifs}+T_{aifs}\\ T_{a,col-a^{\prime },suc}^{\eta \omega }=T_{mu}^{\eta }+T_{atf}^{\omega }+T_{mu}^{\eta }+T_{tb}^{\eta }+T_{mba}^{\eta }+3T_{sifs}+T_{pifs}+T_{aifs}\\ T_{a,col-s^{\prime },suc}^{\eta \omega }=T_{mu}^{\eta }+T_{su}^{\omega }+T_{ba}^{\omega }+T_{sifs}+T_{pifs}+T_{aifs}\\ T_{a,col-s^{\prime },col}^{\eta \omega }=T_{mu}^{\eta }+T_{su}^{\omega }+T_{pifs}+T_{aifs}\\ T_{s,col-a^{\prime },suc}^{\eta \omega }=T_{su}^{\eta }+T_{atf}^{\omega }+T_{mu}^{\eta }+T_{tb}^{\eta }+T_{mba}^{\eta }+3T_{sifs}+T_{pifs}+T_{aifs}\\ T_{s,col-s^{\prime },suc}^{\eta \omega }=T_{su}^{\eta }+T_{su}^{\omega }+T_{ba}^{\omega }+T_{sifs}+T_{pifs}+T_{aifs}\\ T_{s,col-s^{\prime },col}^{\eta \omega }=T_{su}^{\eta }+T_{su}^{\omega }+T_{pifs}+T_{aifs} \end{array}\right.$$

Category ω represents virtual slots where the condition for wide-band transmission is satisfied. As shown in Figure 6, if the starting point of a virtual slot overlaps with the tail end of any narrow-band transmission slot of the BSS pair, a wide-band transmission is allowed for the devices in the target BSS, while the devices in the BSS pair are still captured by the ongoing narrow-band transmission slot. Contrary to this, if the virtual slot of the target BSS overlaps with the sensing period of the BSS pair, all devices in the two BSSs contend the medium equally. Taking this aspect into account, there are seven types of virtual slots in category ω. When the devices in the BSS pair are captured by the ongoing narrow-band transmission, or none of the devices attempt to transmit, the type of the virtual slot is determined by the event of the target BSS. In this case, the virtual slot can have either one of the following events: (1) an idle slot if none of the devices in the target BSS transmit with probability $P_{idle}^{\omega }$, (2) a successful transmission of the target AP with probability $P_{a,suc}^{\omega }$, or (3) the successful transmission of a station in the target BSS with probability $P_{s,suc}^{\omega }$. Given that the BSS pair is in a sensing period, if none of the devices in the target BSS transmit, the virtual slot is occupied by either a successful transmission of the AP pair with probability $P_{a^{\prime },suc}^{\omega }$, or a successful transmission of a station in the BSS pair with probability $P_{s^{\prime },suc}^{\omega }$. Since the AP is configured to initiate a TXOP with an ATF transmission, a virtual slot is occupied by a collision between APs, if none of the stations in the two BSSs have transmitted, except the APs with probability $P_{a,col}^{\omega }$. Otherwise, the event of the virtual slot is a collision among devices including at least one station.

In summary, we have:

(14) $$\left\{ \begin{array}{c} P_{idle}^{\omega }=P_{idle}\left[\phi +\chi P_{idle}\right]\\ P_{a,suc}^{\omega }=P_{a,suc}\left[\phi +\chi P_{idle}\right]\\ P_{s,suc}^{\omega }=P_{s,suc}\left[\phi +\chi P_{idle}\right]\\ P_{a^{\prime },suc}^{\omega }=P_{idle}\chi P_{a,suc}\\ P_{s^{\prime },suc}^{\omega }=P_{idle}\chi P_{s,suc}\\ P_{a,col}^{\omega }=P_{a,suc}\chi P_{a,suc}\\ P_{s,col}^{\omega }=\left(\phi +\chi \right)\left(1-P_{idle}^{\omega }-P_{a,suc}^{\omega }-P_{a,suc}^{\omega }-P_{s,suc}^{\omega }-P_{s,suc}^{\omega }-P_{a,col}^{\omega }\right) \end{array}\right.$$

(15) $$\left\{ \begin{array}{c} T_{idle}^{\omega }=\delta \\ T_{a,suc}^{\omega }=T_{a^{\prime },suc}^{\omega }=T_{atf}^{\omega }+T_{mu}^{\eta }+T_{tb}^{\eta }+T_{mba}^{\eta }+3T_{sifs}+T_{aifs}\\ T_{s,suc}^{\omega }=T_{s^{\prime },suc}^{\omega }=T_{su}^{\omega }+T_{ba}^{\omega }+T_{sifs}+T_{aifs}\\ T_{a,col}^{\omega }=T_{a^{\prime },col}^{\omega }=T_{atf}^{\omega }+T_{aifs}\\ T_{s,col}^{\omega }=T_{s^{\prime },col}^{\omega }=T_{su}^{\omega }+T_{aifs} \end{array}\right.$$

As all possible types of virtual slots are defined, the expected duration of a virtual slot can be given by

(16) $$E\left[T\right]=\sum P^{\eta }T^{\eta }+\sum P^{\eta \omega }T^{\eta \omega }+\sum P^{\omega }T^{\omega }$$

χ is the probability that the BSS pair is observed idle at an arbitrary unit time slot. Therefore, it can be obtained by calculating the expected duration of the idle period over E[T], as follows:

(17) $$\chi =\frac{(P_{idle}^{\eta }+P_{idle}^{\omega })\delta }{E[T]}$$

ϕ can be obtained by taking one unit time slot from each narrow-band transmission event. Therefore,

(18) $$\phi =\frac{\left(1-\chi -\phi \right)\left(P_{a,suc}+P_{s,suc}+P_{a,col}+P_{s,col}\right)\delta }{E[T]}$$

An EDCA transmission may fail due to collision with different sets of devices, depending on whether the devices in the BSS pair are able to access the channel, or with the devices in the two BSSs.

From the perspective of a single device, the EDCA transmission failure probability varies depending on whether the devices in the BSS are able to access the channel, as described in Figure 6. Therefore, the EDCA transmission failure probability of the AP, $P_{a,f}$, and a given station, $P_{s,f}$, in Equation (4) can be obtained as follows:

(19) $$\left\{ \begin{array}{c} \begin{array}{cc} \begin{array}{c} P_{a,f}= \end{array} & \tau_{a}\left[\left(1-\chi \right)\left[1-{\left(1-\tau_{s}\right)}^{N}\right]+\chi \left[1-\left(1-\tau_{a^{\prime }}\right){\left(1-\tau_{s}\right)}^{N}{\left(1-\tau_{s^{\prime }}\right)}^{N^{\prime }}\right]\right]\\ P_{s,f}= & \tau_{s}\left[\left(1-\chi \right)\left[1-\left(1-\tau_{a}\right){\left(1-\tau_{s}\right)}^{N-1}\right]+\chi \left[1-\left(1-\tau_{a}\right)\left(1-\tau_{a^{\prime }}\right){\left(1-\tau_{s}\right)}^{N-1}{\left(1-\tau_{s^{\prime }}\right)}^{N^{\prime }}\right]\right] \end{array} \end{array}\right.$$

Now, it is possible to obtain the throughput of the devices. APs transmit multiplexed data whenever a TXOP is acquired or is triggered by the AP pair. Let *M* be the multiplexing size. Then, the throughput of a single AP, $E[S_{a}]$, can be obtained by recording all successful transmission events of the target AP, as well as all successful wide-band transmission events of the AP pair. Therefore, we have:

(20) $$\begin{array}{cc} E[S_{a}]= & \frac{M\cdot l}{E[T]}(P_{a,suc}^{\eta }+2P_{a,suc-a^{\prime }suc}^{\eta \omega }+P_{a,suc-s^{\prime }suc}^{\eta \omega }+P_{a,suc-s^{\prime }col}^{\eta \omega }\\ & +P_{a,col-a^{\prime }suc}^{\eta \omega }+P_{s,suc-a^{\prime }suc}^{\eta \omega }+P_{s,col-a^{\prime }suc}^{\eta \omega }+P_{a,suc}^{\omega }+P_{a^{\prime },suc}^{\omega }) \end{array}$$

In the proposed scheme, a station transmits data by acquiring a TXOP or when triggered by an AP. If a round-robin scheduler is assumed, the probability that a station is scheduled for an MU cascading sequence of the AP is M/N. Therefore, the throughput of a single station, $E[S_{s}]$, is

(21) $$\begin{array}{cc} E[S_{s}]= & \frac{M}{N}\frac{l}{E[T]}(P_{a,suc}^{\eta }+2P_{a,suc-a^{\prime }suc}^{\eta \omega }+P_{a,suc-s^{\prime }suc}^{\eta \omega }+P_{a,suc-s^{\prime }col}^{\eta \omega }\\ & +P_{a,col-a^{\prime }suc}^{\eta \omega }+P_{s,suc-a^{\prime }suc}^{\eta \omega }+P_{s,col-a^{\prime }suc}^{\eta \omega }+P_{a,suc}^{\omega }+P_{a^{\prime },suc}^{\omega })\\ & +\frac{l}{E[T]}\left(P_{s,suc}^{\eta }+P_{s,suc-a^{\prime }suc}^{\eta \omega }+P_{s,suc-s^{\prime }suc}^{\eta \omega }+P_{s,suc-s^{\prime }col}^{\eta \omega }+P_{s,suc}^{\omega }\right) \end{array}$$

Finally, the system throughput of the target BSS can be obtained as follows:

(22) $$E\left[S\right]=E\left[S_{a}\right]+N\cdot E\left[S_{s}\right]$$

In this section, the analysis models the complete set of the proposed scheme which includes multi-AP coordinated transmission, MU cascading and MU EDCA. However, the analysis can also be applied to different combinations of each feature by modifying a part of parameters in this section. For example, if MU EDCA is excluded, STA and AP have the same transmission probability for a virtual slot, which is

(23) $$\tau_{a}=\tau_{s}=\tau$$

In case of excluding MU cascading, a trigger frame is no longer be piggybacked in the DL MU PPDU of AP, and the transmission sequence initiated by an AP is terminated with an acknowledgement frame transmitted by stations. Therefore, the time duration of each individual virtual slot in Equations (9) and (24) can be modified as follows:

(24) $$\left\{ \begin{array}{c} T_{idle}^{\eta }=\delta \\ T_{a,suc}^{\eta }=T_{mu}^{\eta }+T_{mba}^{\eta }+T_{sifs}+T_{aifs}\\ T_{a,suc}^{\omega }=T_{a^{\prime },suc}^{\omega }=T_{atf}^{\omega }+T_{mu}^{\eta }+T_{mba}^{\eta }+2T_{sifs}+T_{aifs}\\ T_{s,suc}^{\omega }=T_{s^{\prime },suc}^{\omega }=T_{su}^{\omega }+T_{ba}^{\omega }+T_{sifs}+T_{aifs}\\ T_{a,col}^{\omega }=T_{a^{\prime },col}^{\omega }=T_{atf}^{\omega }+T_{aifs}\\ T_{s,col}^{\omega }=T_{s^{\prime },col}^{\omega }=T_{su}^{\omega }+T_{aifs} \end{array}\right.$$

## 4. Performance Evaluation

In this section, we discuss the performance evaluation of the proposed scheme. An event-driven MAC-level simulator described in [14] is used for the evaluation. The simulator was developed in MATLAB, which implements IEEE 802.11 EDCA functions and IEEE 802.11ax TUA functions based on the proposed physical layer numerology of IEEE 802.11be discussed in Section 2. In order to focus on the MAC level transmission efficiency of the proposed scheme, ideal channel conditions (no channel errors, hidden terminals, or capture) is assumed for the simulation.

In the simulation, a topology with two IEEE 802.11be BSSs is considered, as illustrated in Figure 1. Each BSS comprises one IEEE 802.11be AP, and a variable number of IEEE 802.11be stations. The system parameters are listed in Table 1. In the simulation, it is assumed that the OFDMA numerology and the preamble formats of IEEE 802.11 are the same as those in IEEE 802.11ax. Given this, we compared the performance of the following four schemes. (1) legacy transmission (DLMU): In this scheme, a multi-AP coordinated OFDMA transmission does not occur to demonstrate the conventional environment. At the beginning of each TXOP, the TXOP holder device performs wide-band transmission whenever possible. For pair comparison with the proposed schemes in terms of downlink throughput, the AP performs a DL MU OFDMA transmission in its TXOP, using the 11ax MU PPDU format. The TXOP ends with UL MU multi-STA block ACK (MBA) transmission of the recipient stations. (2) Coordinated OFDMA (CO): In this scheme, the proposed multi-AP coordinated OFDMA is enabled without MU cascading and MU EDCA. Therefore, whenever wide-band transmission is allowed, the AP transmits an ATF to the AP pair to share its TXOP, and each AP transmits a narrow-band DL MU PPDU from its primary channel. The transmission sequence is terminated by the UL MU MBA transmission of the recipient stations. (3) Coordinated OFDMA with MU cascading (CO+CA): In this scheme, the AP always initiates an MU cascading sequence when it obtains a TXOP, or when it is triggered by the pair AP. Therefore, in response to the TFs aggregated in a DL MU transmission of the AP, all recipient stations transmit uplink data in the TB PPDU format. The transmission sequence terminates when the AP performs the DL MBA transmission. (4) Coordinated OFDMA with MU cascading and MU EDCA (CO+CA+ME): This scheme includes all features introduced in Section 2. The transmission sequence of the AP is identical to that of CO+CA, but the difference is that all recipient stations need to apply the MU EDCA parameter set after UL MU transmission. In this simulation, the MU EDCA minimum CW size is set by multiplying the minimum contention window size ($CW_{min}$) by an MU EDCA multiplier (α).

**Figure 8:**
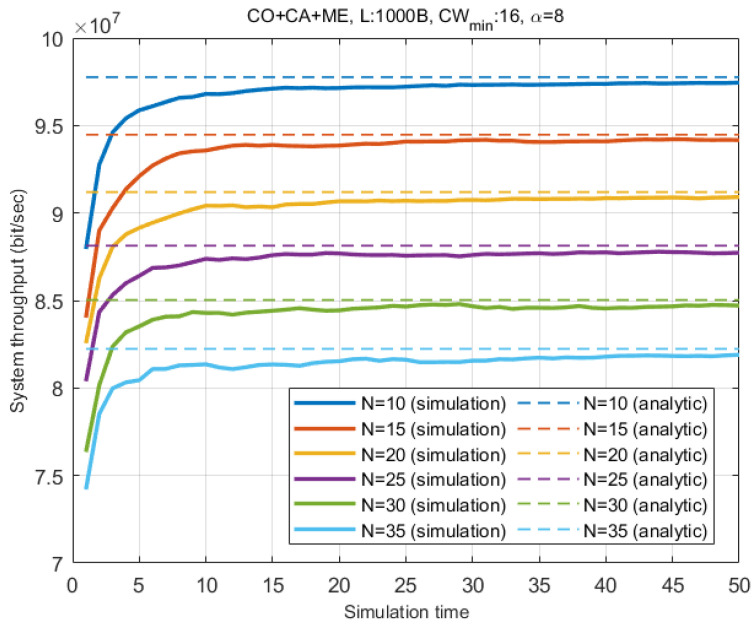
System throughput over time.

In order to validate the analysis model in Section 3, the analytic results and the simulation results are plotted in Figure 8. Since we assume ideal physical channel and there is no BSS other than the target two BSSs, once a wide-band transmission succeeds, virtual slot events and wide-band channel sensing status are synchronized for all stations. In this case, ϕ and χ become zero and one, respectively, in Section 3, and wide-band events, ω, dominates the system throughput. The figures indicate that the analytic results of our proposed model are closely matched with the simulation results.

**Figure 9:**
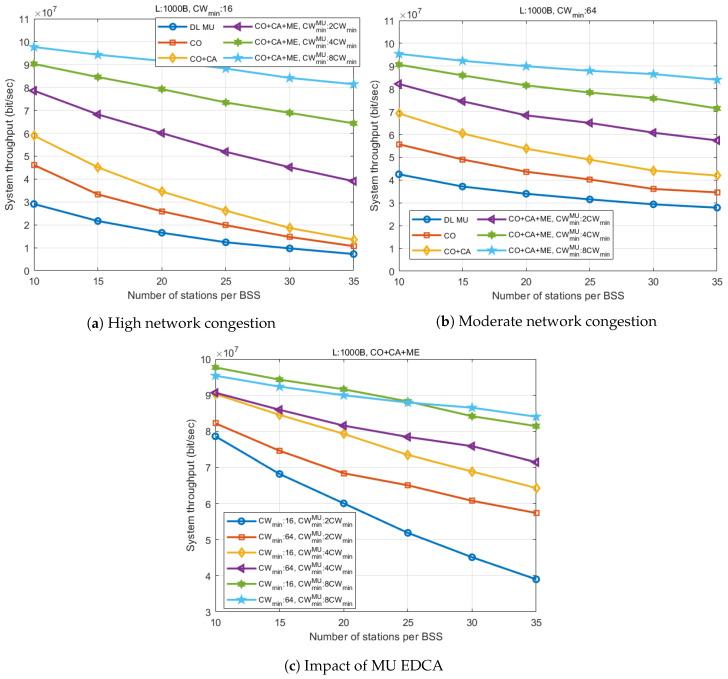
System throughput comparison.

Figure 9 presents a comparison of the system throughput of each scheme for different numbers of associated stations.

Figure 9a shows that the proposed coordinated OFDMA (CO) provides a 53% throughput gain over the legacy scheme (DLMU) for a small number of stations (N=10). By applying MU cascading, an additional 27% gain is achieved over CO (96% over DLMU), as additional uplink data is aggregated in the TXOPs of the AP. The gain from CO and CA tends to decrease as the number of stations increases. In the figure, a larger number of stations demonstrate the environment where the contention window does not handle the network congestion sufficiently, resulting in an increase in collisions. In such cases, the coordinated OFDMA combined with MU cascading and MU EDCA (CO+CA+ME) exhibits remarkable throughput enhancement. By effectively suppressing the channel access intensity of stations, CO+CA+ME realizes a 181% gain over CO+CA in a dense network environment (N=35), even with the smallest MU EDCA multiplier (α=2). The throughput gap becomes greater as α increases, as the throughput of other schemes without MU EDCA collapse is affected by the network congestion.

Figure 9b demonstrates a situation where the network congestion is properly controlled by greater contention window size. In this case, as shown in the figure, the gain can be distinguished according to the addition of each feature. In a low station density environment (N=10), the system throughput increases by approximately $1.4\times 10^{7}$ Mbps as each feature is added. While the slope of each graph appears similar, it is shown that the proposed scheme (CO+CA+ME) realizes the greatest throughput in all ranges of the number of stations, by improving the throughput at most $5.6\times 10^{7}$ Mbps (α=8) compared to DLMU. However, the simulation result is based on the assumption that the buffer of all stations is full. Therefore, in real circumstances, the throughput of the proposed scheme may decrease depending on the scheduling efficiency of the AP. If the stations have a heavy traffic load, it is hard to expect the AP to perform UL MU scheduling for newly generated uplink traffic of stations immediately. Furthermore, the scheduling efficiency of the 802.11ax AP is highly dependent on the buffer status report of each station. Thus, it may not be desirable to set an excessively large MU EDCA parameter considering latency, and to keep stations from sending a buffer status report autonomously. Maintaining the scheduling efficiency without having an EDCA channel access of station may be possible upon incorporating an advanced feature, such as the null data packet feedback report (NFR) procedure, that triggers a buffer status report of a large number of stations based on TUA, as part of a cascading sequence.

Figure 9c presents a comparison of the system throughput performance of the proposed scheme (CO+CA+ME) according to the change in the minimum contention window size, and the MU EDCA multiplier (α). The result shows that the system throughput increases with α, regardless of the value of the minimum contention window size. For the greatest α (α=8), the throughput performance of the two different minimum CW sizes has almost the same slope. However, the case with a larger $CW_{min}$ has a slightly lower throughput in the range of a small number of stations, and the results reversed as the number of stations increased. This is because an excessively large $CW_{min}$ is set above a level sufficient to avoid collision, causing unnecessary deferring delay in AP transmission. However, as the number of stations increases, collisions start to affect the system throughput. As smaller values of α, collisions start to affect the throughput, the performance gap between two graphs with the same α value increases as α decreases.

**Figure 10:**
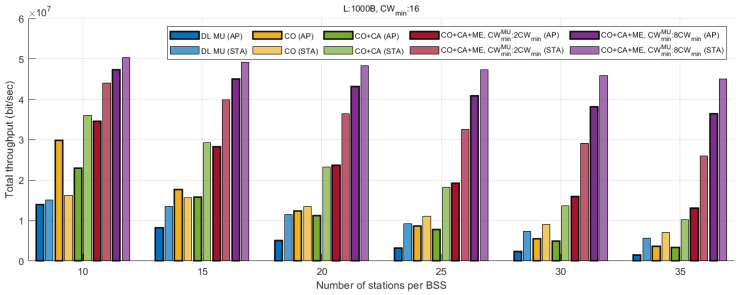
Comparison of downlink/uplink throughput.

Figure 10 presents a comparison of the sum of the UL and DL throughput of each scheme. According to the figure, CO increases the downlink throughput by up to 173%. One observation is that the total uplink throughput slightly increases as well, due to the decrease in the collision overhead. If an wide-band transmission collides, the TXOP of the corresponding AP is released immediately after the transmission of ATF, where the air-time is much shorter than that for DL MU PPDU. As a result, the overall channel utilization is improved. As CA is additionally applied, the UL throughput increases considerably in all station ranges due to additional TUA transmission opportunities. When the number of stations is 10, where the probability of collision is relatively low, additional aggregation of UL MU data reduces the total downlink throughput. This is because the increased AP TXOP duration reduces transmission opportunities. However, as the number of stations increases and collision overhead determines the throughput performance, the DL performance gap between the two schemes, CO and CO+CA, appears to be almost the same. As ME is applied, the throughput of both DL and UL increases significantly, especially for greater α values. Compared with other schemes, CO+CA+ME is shown to have less throughput loss due to increased network congestion. While DLMU exhibits 88% degradation of the DL throughput as the number of stations increases from 10 to 35, CO+CA+ME (α=8) exhibits only 23% degradation. In this result, although the UL throughput is higher than the DL throughput, a major portion of the uplink throughput is generated by the MU cascading sequences of the AP. In an MU cascading sequence, the AP can suppress UL flows by reducing the size of the triggered data. Since all UL flows converge to the AP, it can adaptively manage the UL and DL flow, depending on the network condition based on the traffic statistics.

**Figure 11:**
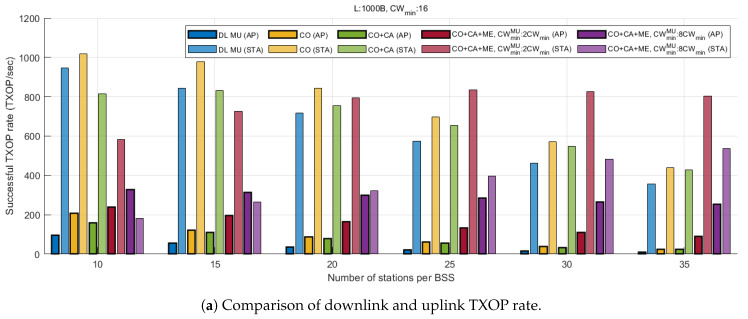

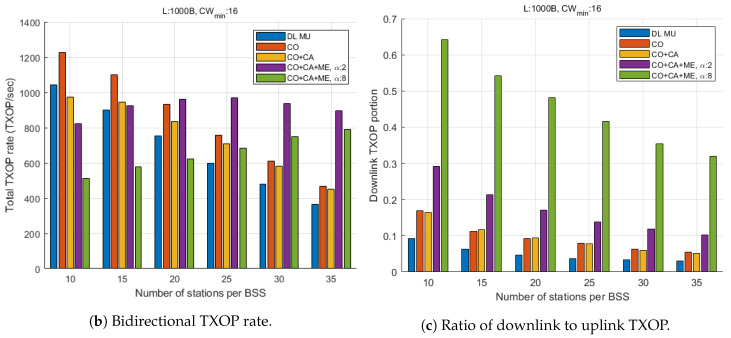
TXOP statistics.

Figure 11 displays various statistics corresponding to the TXOP from the simulation. Among the results, only successful TXOPs in which the entire transmission sequence ends properly without a collision are considered. For DL TXOP, a successful transmission triggered by an ATF is considered as a TXOP of the triggered AP, because the triggered AP can exclusively use the assigned resource including, for example, the scheduling and the duration of DL and UL transmission. For UL TXOPs, all successful EDCA UL transmissions performed by the associated stations of the target BSS are counted. Figure 11a compares the TXOP rate of the AP and the stations. The result shows that for the conventional scheme (DLMU), AP encounters a severe TXOP unfairness as the number of stations increases. Since we assumed saturated traffic, all devices in the same BSS as the AP have an equal channel access probability for each virtual slot, when applying the same EDCA parameters. In the case of DLMU, the DL TXOP rate is inversely proportional to the number of stations in the BSS, which causes the starvation of the AP. As shown in Figure 11c, the proportion of DL TXOP is 0.9 for 10 stations and 0.029 for the other 35 stations. As coordinated OFMDA is applied (CO), the DL TXOP portion doubles across all station ranges, and as shown in Figure 11b, the total TXOP rate also increases because of improved channel utilization, compared with DLMU. With the addition of MU cascading (CO+CA), the total TXOP rate decreased with the number of stations, owing to the increased length of AP TXOP. However, as the station density increases and collision overhead dominates the system performance, the rate becomes similar to that of CO. In terms of the fairness between the DL and UL TXOP, MU cascading does not exhibit significant improvements over CO. However, the importance of MU cascading lies in applying MU EDCA to stations through TUA. MU EDCA (CO+CA+ME) not only yields a significant improvement in the DL TXOP rate, but also mitigates performance degradation with increasing station density, in comparison with other schemes. For instance, the portion of DL TXOP accounts for more than 10% (α=2) or 32% (α=8) of the total, even when the number of stations is 35. The results show that the proposed scheme guarantees enhanced DL/UL fairness, regardless of the level of network congestion. In addition, it is expected that the DL/UL flows can be further optimized in various ways, depending on the traffic conditions and requirements, by manipulating α and the size of the UL triggered data.

## 5. Conclusions

In this paper, we introduced a novel multi-AP cooperative transmission scheme for IEEE 802.11be networks.

Utilizing the concept of multi-AP coordinated OFDMA of IEEE 802.11be, and considering advanced features of IEEE 802.11ax, such as TUA, MU cascading sequence, and MU EDCA, the proposed transmission scheme allows an AP to share a granted TXOP with nearby APs, to ensure that APs can have improved control over the medium.

In addition, we presented a mathematical model for analyzing the delay characteristics of the proposed scheme, using Markov chain analysis. The simulation results proved that the system throughput of the proposed scheme is better than that of the conventional DL OFMDA of IEEE 802.11ax, as the proposed coordinated transmission scheme effectively allows APs to have increased transmission opportunities. Applying MU EDCA and MU cascading, the proposed scheme guarantees not only better robustness against network congestion, but also improved flow control between the UL/DL traffic.

The proposed transmission scheme is carefully designed to be fully compatible with the conventional IEEE 802.11 protocols, to ensure that implementation changes can be minimized. Therefore, the proposed scheme is potentially universally applicable.

## Figures and Tables

**Table 1:** Simulation parameters.

Parameter	Value
Number of BSSs	2
Number of stations per BSS	10, 15, 20, 25, 30, 35
Transmission bandwidth	20, 40 MHz
11p data subcarrier	242, 484 subcarriers
11p OFDM symbol duration	16 μs
Number of RUs for MU transmission	9 RUs
11be OFDM symbol duration	16 μs
Legacy preamble duration	40 μs
11be SU PPDU preamble duration	48 μs
11be MU PPDU preamble duration	48 μs
11be TB PPDU preamble duration	48 μs
MCS	7
Trigger frame MAC payload	38 Bytes
AP trigger frame MAC payload	38 Bytes
MAC data payload (*L*)	1000 Bytes
Slottime	9 μs
SIFS	16 μs
PIFS	25 μs
AIFS	34 μs
Minimum contention window size ($CW_{min}$)	16, 64
MU EDCA multiplier (α)	2, 4, 8
MU EDCA minimum contention window size	$\alpha CW_{min}$
MU EDCA timer	1 s
Simulation duration	50 s

## References

[1] Telecom Advisory Services, LLC (2018). The Economic Value of Wi-Fi: A Global View (2018 and 2023). https://www.wi-fi.org/file/the-economic-value-of-wi-fi-a-global-view-2018-and-2023.

[2] Cisco (2019). Cisco Annual Internet Report (2018–2023) White Paper. https://www.cisco.com/c/en/us/solutions/collateral/executive-perspectives/annual-internet-report/white-paper-c11-741490.html.

[3] Bianchi G. (2000). Performance analysis of the IEEE 802.11 distributed coordination function. IEEE J. Sel. Areas Commun..

[4] Katayama Y., Umehara D., Wakasugi K. A mathematical model of access control to allocate downlink bandwidth in high-density wireless LAN. Proceedings of the 2017 11th International Conference on Signal Processing and Communication Systems (ICSPCS).

[5] Lim W.S., Kim D.W., Suh Y.J. (2011). Achieving fairness between uplink and downlink flows in error-prone WLANs. IEEE Commun. Lett..

[6] Kim J., Kim S.H., Sung D.K. (2018). Hybrid ARQ-based uplink/downlink fairness enhancement in WLAN. IEEE Trans. Veh. Technol..

[7] IEEE (2017). P802.11ax, Project Authorization Request. IEEE Standard Association. https://standards.ieee.org/project/802_11ax.html.

[8] IEEE (2019). P802.11be, Project Authorization Request. IEEE Standard Association. https://standards.ieee.org/project/802_11be.html.

[9] Au E. (2019). IEEE 802.11 be: Extremely High Throughput [Standards]. IEEE Veh. Technol. Mag..

[10] Clifford P., Duffy K., Leith D.J., Malone D. On improving voice capacity in 802.11 infrastructure networks. Proceedings of the 2005 International Conference on Wireless Networks, Communications and Mobile Computing.

[11] (2013). IEEE Standard for Information Technology-Telecommunications and Information Exchange Between Systems Local And Metropolitan Area Networks Specific Requirements Part 11: Wireless LAN Medium Access Control (MAC) and Physical Layer (PHY) Specifications Amendment 4: Enhancements for Very High Throughput for Operation in Bands below 6 GHz. IEEE Std. 802.11ac-2013.

[12] IEEE (2019). Draft Standard for Information Technology—Telecommunications and Information Exchange between Systems Local and Metropolitan Area Networks—Specific Requirements, Part 11: Wireless LAN Medium Access Control (MAC) and Physical Layer (PHY) Specifications, Amendment 1: Enhancements for High Efficiency WLAN. IEEE P802.11ax^TM^/D5.1.

[13] Lee J., Kim W., Lee S.J., Jo D., Ryu J., Kwon T., Choi Y. An experimental study on the capture effect in 802.11a networks. Proceedings of the 2nd ACM International Workshop on Wireless Network Testbeds, Experimental Evaluation and Characterization, WiNTECH ’07, Co-located with the ACM MobiCom 2007 Conference.

[14] Ahn W., Kim R.Y. (2019). Distributed Triggered Access for BSM Dissemination in 802.11bd V2V Networks. Appl. Sci..

